# Role of Clinical and Multimodality Neuroimaging in the Evaluation of Brain Death/Death by Neurologic Criteria and Recent Highlights from 2023 Updated Guidelines

**DOI:** 10.3390/diagnostics14121287

**Published:** 2024-06-18

**Authors:** Pokhraj Prakashchandra Suthar, Miral D. Jhaveri, Avin Kounsal, Lillian D. Pierce, Jagadeesh S. Singh

**Affiliations:** Department of Diagnostic Radiology & Nuclear Medicine, Rush University Medical Center, Chicago, IL 60612, USA; miral_d_jhaveri@rush.edu (M.D.J.); avin_kounsal@rush.edu (A.K.); lillian_d_pierce@rush.edu (L.D.P.); jagadeesh_s_singh@rush.edu (J.S.S.)

**Keywords:** brain death, clinical diagnosis, pathophysiology, imaging

## Abstract

*Purpose of Review:* This review aims to provide a comprehensive overview of the diagnosis of brain death/death by neurologic criteria (BD/DNC) by emphasizing the clinical criteria established by the American Academy of Neurology (AAN) in light of their updated guidelines released in 2023. In this review, we will focus on the current implementation of ancillary tests including the catheter cerebral angiogram, nuclear scintigraphy, and transcranial Doppler, which provide support in diagnoses when clinical examination and apnea tests are inconclusive. Finally, we will also provide examples to discuss the implementation of certain imaging studies in the context of diagnosing BD/DNC. *Recent Findings:* Recent developments in the field of neurology have emphasized the importance of clinical criteria for diagnosing BD/DNC, with the AAN providing clear updated guidelines that include coma, apnea, and the absence of brainstem reflexes. Current ancillary tests, including the catheter cerebral angiogram, nuclear scintigraphy, and transcranial Doppler play a crucial role in confirming BD/DNC when the clinical assessment is limited. The role of commonly used imaging studies including computed tomography and magnetic resonance angiographies of the brain as well as CT/MR perfusion studies will also be discussed in the context of these new guidelines. *Summary:* BD/DNC represents the permanent cessation of brain functions, including the brainstem. This review article provides the historical context, clinical criteria, and pathophysiology that goes into making this diagnosis. Additionally, it explores the various ancillary tests and selected imaging studies that are currently used to diagnose BD/DNC under the newly updated AAN guidelines. Understanding the evolution of how to effectively use these diagnostic tools is crucial for healthcare professionals who encounter these BD/DNC cases in their practice.

## 1. Introduction

Brain death/death by neurologic criteria (BD/DNC) is a permanent cessation of brain function, including brainstem reflexes and circulatory and respiratory functions [1]. This results from devastating brain injuries secondary to head trauma, stroke, intracranial bleeding, or following cardiac arrest. Furthermore, 2.06% of deaths in the United States are accounted for by this diagnosis [2,3]. With the recent advancement of medical services in which respiratory and circulatory functions can be supported by external life support, the declaration of BD/DNC is now associated with ethical and legal controversies. Therefore, the timely and accurate diagnosis of BD/DNC is crucial in the prevention of unnecessary medical interventions and in expediting organ transplantation whenever possible [3]. The American Academy of Neurology (AAN) has provided guidelines for the clinical criteria for the diagnosis of BD/DNC [4,5]. An overview of the usage of the necessary ancillary tests required to support this diagnosis when an apnea test or neurological exam is not sufficient is also outlined. These guidelines were recently updated in October of 2023 and now incorporate both pediatric and adult patients [4,5]. These updates and their implications for the corresponding ancillary tests as well as specific examples of certain additional imaging studies will be discussed in this article.

## 2. History

In 1969, Mollaret and Goulon introduced the concept of brain death secondary to brain damage as the deepest form of coma without the possibility of recovery [6]. In 1968, the Ad Hoc Committee of Harvard Medical School described irreversible coma and gave this neurological state a new definition of death called brain death [7]. In 1981, the President’s Commission for the Study of Ethical Problems in Medicine and Behavioral Research issued a report on “Defining Death”, and their criteria for the diagnosis of brain death in adults include the complete, irreversible cessation of all functions of the entire brain, referred to then as a “whole brain concept” [8].

In 1981, the National Conference of Commissioners on Uniform State Laws implemented the “Uniform Determination of Death Act (UDDA)” for a uniform law on “brain death” throughout the nation, and it was approved by the American Medical Association and American Bar Association [9]. However, the UDDA did not define the neurological criteria, which diagnostic tests were needed, and the “accepted medical standards” [8,10]. In 1994, it was the AAN that attempted to standardize the neurologic criteria and practice parameters for determining brain death as it has evolved. The original guidelines were published in 1995 and updated in 2010, with the most recent update occurring last year in 2023 [4,5,9]. Of note, the newest guidelines use the term “brain death/death by neurologic criteria” (or “BD/DNC”) to discuss the permanent loss of function, which we shall be using to refer to the criteria discussed in this article [5]. The 2020 report by Greer DM and colleagues from the World Brain Death Project outlines the minimum clinical standards for determining brain death or death by neurologic criteria in both adults and children, offering clear guidance for different clinical scenarios. These recommendations, endorsed by numerous international societies, can assist professional organizations and countries in revising or creating protocols and procedures for brain death determination, promoting greater consistency within and between nations [11].

## 3. Clinical Criteria and Declaration of Brain Death/Death by Neurologic Criteria (BD/DNC) in Light of the New 2023 AAN Guidelines

Certain clinical factors and diagnoses should be assessed and considered prior to the declaration of BD/DNC. The new AAN guidelines provide prerequisites regarding the appropriate determination of this declaration and now include both pediatric (>37 weeks corrected gestational age) and adult patients [5]. Patients with an appropriate traumatic brain etiology first need to be evaluated for any level of consciousness, brainstem reflex or motor preservation, or spontaneous breathing, which would exclude them from further diagnostic examination for BD/DNC. Per the current guidelines, the pathophysiology of brain injury mainly dictates how long a patient may need to be observed prior to initiating BD/DNC evaluation. 

## 4. A Brief Overview of Clinical and Laboratory Criteria

Once the appropriate etiology of brain injury has been established, certain factors that mimic and confound BD/DNC should also be assessed for and excluded, including hypothermia, drug usage, and electrolyte imbalances. Hypothermia is one of the mimics of BD/DNC, and sufficient time for observation in the setting of hypothermia as well as drug usage is needed to effectively remove these confounders upon evaluation [12,13]. An observation time of at least 24 h is now needed after returning the patient’s core temperature to >36 degrees Celsius when the patient presents in a hypothermic state. Regarding drug usage, if the offending drug is known, it is recommended that the patient is observed for a minimum of five half-lives after drug discontinuation prior to BD/DNC assessment [14]. Additional guidelines which are more specific regarding phenobarbital blood levels, pharmacologic analysis, and indicated screening blood and urine tests are also now provided. Lab values including blood electrolytes and glucose must also be corrected prior to assessment, and additional details on confounding laboratory derangements are provided in the new guidelines. According to the new guidelines, pregnancy is not a contraindication for the evaluation of BD/DNC.

The assessment for BD/DNC necessitates a comprehensive evaluation encompassing unresponsiveness to visual, auditory, and tactile stimuli, as well as the absence of motor responses in facial features and extremities, excluding spinal reflexes. Additionally, the absence of pupillary, oculocephalic, oculovestibular, corneal, gag, and cough reflexes should be meticulously assessed, with particular consideration given to the cervical spine and skull base integrity. In infants younger than six months, the absence of sucking and rooting reflexes is also indicative. All components of the examination must align with the criteria for BD/DNC for a conclusive determination, emphasizing the importance of thorough and consistent evaluation [5]. 

Finally, an apnea test is also performed. At the time of testing, the patient should [5,15,16,17] meet the following criteria:

- Be normothermic (core temperature of ≥36.5 degrees Celsius);
- Be hemodynamically stable (systolic blood pressure ≥100 mm Hg and mean arterial pressure ≥75 mm hg in adults, or, recently added in the new guidelines, a target blood pressure close to patient’s known chronic baseline if available);
- Have a confirmed absence of sedative and paralytic drugs;
- Have normal oxygenation (PaO_2_ ≥ 200 mmHg after 100% oxygenation);
- Have near-normal PaCO_2_ (35–45 mmHg), unless the patient is known to be hypercarbic;
- As per the updated 2023 guidelines, have an arterial pH of 7.35–7.45.

No evidence of respiratory effort and a rise in the blood carbon dioxide level of >60 mm Hg, 20 mm Hg from the baseline, and an arterial pH < 7.3 after 10 min of removing a patient from mechanical ventilation is considered a positive apnea test [5,15]. A rise in the blood carbon monoxide level of <60 mm Hg or <20 mm Hg from the baseline is considered an indeterminate or inconclusive apnea test. 

Ancillary testing is required to support the diagnosis of BD/DNC when neurological examinations and apnea testing cannot be completed, the findings are indeterminate, or the patient has persistent metabolic derangements on laboratory studies despite findings which are consistent with BD/DNC per the current AAN recommendations. The catheter cerebral angiogram, nuclear scintigraphy, and transcranial Doppler ultrasonography are recommended as ancillary tests for BD/DNC diagnosis [18] (Table 1). As of the 2023 AAN guideline updates, electroencephalography and auditory-evoked potentials are no longer considered ancillary tests, and transcranial Doppler ultrasonography is only appropriate in adult patients. Most of the United States and many countries worldwide use the AAN clinical criteria for BD/DNC diagnosis, though variability persists in the preclinical testing, clinical examination, ancillary tests, and apnea tests [15,19,20]. The new AAN guidelines continue to try to address these discrepancies, some of which we have discussed or will discuss in this article. However, legal variability is still present in various countries, and ancillary tests are sometimes mandatory. 

## 5. Declaration of the Brain Death/Death by Neurologic Criteria (BD/DNC)

Most of the United States legally allows appropriately licensed physicians to determine BD/DNC; however, updates to examiner qualifications have been made, and trainees may be supervised as needed in settings where they are not permitted to examine these patients independently [21]. There is a comprehensive protocol for assessing the BD/DNC (Table 2). The guidelines now include both pediatric (patients > 37 weeks gestational age) and adult patients, with a few additional differences between the assessment of the two groups [5]. A single examination including the apnea test is the minimum standard for BD/DNC in adult patients [15]. Two assessments, a minimum of 12 h apart, are now required in pediatric patients. After cardiopulmonary resuscitations or other severe acute brain injuries, the assessment of BD/DNC should be deferred for 24 h or longer if there are inconsistencies in the original examination [22]. The Organ Procurement Organization should be notified about a plan to evaluate for BD/DNC, and they should be available to provide grief counseling and information to the family regarding organ donation [19,23]. Organ support including the maintenance of adequate ventilation and pressure is continued after the declaration of BD/DNC if the patient is a potential candidate for organ donation. Organ support is also continued in pregnant patients to support the fetus [15]. There is an urgent unmet need for the continuous review and adherence to institutional standards for determining death by neurologic criteria (DNC). Both referring hospitals and organ procurement organizations (OPOs) share a responsibility for preventing errors in DNC before organ recovery [24].

The catheter cerebral angiogram, nuclear scintigraphy, and transcranial Doppler are recommended ancillary tests for the diagnosis of BD/DNC by the AAN, as will be discussed further below. Additionally, frequently used imaging techniques such as a computed tomography angiogram (CTA), magnetic resonance angiogram (MRA), and CT/MR perfusion methods are not recommended by the AAN for diagnosis, but may be used as supplemental imaging to further evaluate the clinical findings we have discussed thus far [15]. The neuroimaging findings regarding the patients may be used as a prerequisite for BD/DNC evaluation. This is why radiologists play such a critical role in the diagnosis of early hypoxic-ischemic changes in the brain and brainstem and provide potential supplemental guidance, such as the exclusion of mimicking BD/DNC diagnoses, in the neurologist’s clinical evaluation of BD/DNC.

## 6. Pathophysiology of BD/DNC

Extensive brain injury from trauma, stroke, subarachnoid hemorrhage, and cardiopulmonary arrest causes a combination of hypoxic and ischemic brain injury, leading to cytotoxic and vasogenic edema and an increase in intra-cranial pressure (ICP). A normal cerebral blood flow (CBF) at rest is 45–60 mL/100 gm of brain tissue per minute. The CBF is regulated by the cerebral perfusion pressure (CPP) and cerebrovascular resistance (CVR). CPP is the difference between the mean arterial pressure (MAP) and ICP [25].

CBF = CPP/CVR = MAP − ICP/CVR

The skull limits the intracranial volume, and an increase in intracranial hypertension causes a decrease in the CPP. However, brain autoregulation is activated and maintains the CBF by decreasing the vessel tone, leading to a decreased CVR. This mechanism can preserve the cerebral blood flow up to 50 mmHg CPP. Brain autoregulation fails when a further decrease in the CPP leads to a decreased CBF. The end-diastolic cerebral flow completely ceases as the ICP reaches the diastolic pressure; at this stage, the CBF is significantly reduced but is still able to preserve the viability of brain parenchyma. Brain circulatory arrest occurs when the ICP exceeds the diastolic pressure of the MAP and leads to the compression of the cerebral capillaries and venules. Large major cerebral arteries are still patent, and the systolic phase of the heartbeat causes the distension of elastic walls of major cerebral arteries and tries to maintain the antegrade blood flow. However, during diastole, the same blood volume has a retrograded flow due to the elastic recoil. These oscillating to-and-fro movements cause a net zero cerebral blood flow and lead to the death of neurons in 10 to 15 min. Blood movement in the cerebral artery ceases completely when the ICP exceeds the systolic pressure, but oscillation can still be seen in extracranial segments of the internal carotid and vertebral arteries (Table 3).

## 7. Neuroimaging Technique for BD/DNC

### 7.1. Nuclear Scintigraphy 

The Society of Nuclear Medicine (SNM) published the first BD/DNC scintigraphy guideline in 2003 and revised it in 2012 [26,27]. BD/DNC scintigraphy is indicated for assessing the brain flow in suspected patients with BD/DNC [28]. It is 100% sensitive and 100% specific for diagnosing BD/DNC [29,30,31]. 

The patient should have a stable blood pressure and be normally ventilated to prevent changes in the cerebral blood flow caused by hyperventilation. In some institutes, a tourniquet is placed in the head above the eyebrows, ear, and around the occipital prominence of the skull to immobilize the head, and it helps to decrease the scalp blood flow during scanning. However, the tourniquet should be avoided in patients with a head injury. 

Either brain specific agents, ^99m^Tc-ECD (99m Technetium Ethyl cysteinate dimer), ^99m^Tc-HMPAO (99m Technetium Hexamethylpropyelene amine oxime), or non-brain specific/flow agents such as ^99m^Tc-DTPA (99m Technetium Diethylenetriaminepentaacetic acid) can be used for brain scintigraphy [32].

Brain-specific agents are lipophilic, cross the BBB, and are accumulated proportional to the blood flow in normal gray matter, including brain cells of the cerebrum, cerebellum, and brainstem [32]. Therefore, in addition to the flow, brain parenchyma is also seen in the normal functioning brain. On the other hand, flow agents like ^99m^Tc-DTPA cannot cross the blood–brain barrier, providing information on the low-resolution vascular flow only with a lack of parenchymal perfusion; hence, brain-specific agents are preferred [33,34]. The usual injected radiopharmaceutical dose is 30 mCi (1110 MBq) in adults, and the pediatric dose is calculated based on body weight (0.3 mCi/kg, and minimum dose of 5 mCi (185 MBq)) for brain-specific agents [34].

**Figure 1:**
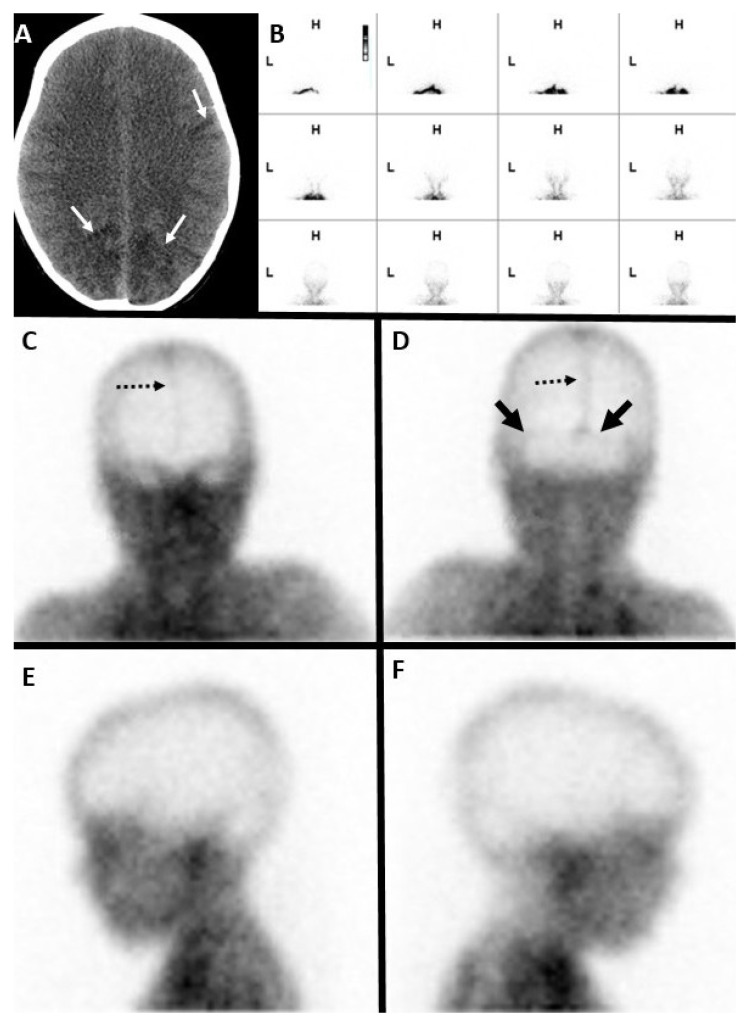
Seven-year-old male with a hypoxic-ischemic injury. (**A**) Unenhanced axial CT brain in the brain window demonstrates bilateral occipital and left fronto-parietal lobes (white arrows) ischemic infarct in the hypoxic injury. A Tc-99m DTPA nuclear scintigraphy study after the injection of 11.1 mCi of Tc-99m DTPA. (**B**) Initial blood flow phase image series at 2 s intervals. (**C**–**F**) Delayed static images at 20 min after tracer injection in (**C**) anterior, (**D**) posterior, (**E**) right, and (**F**) left lateral projections. Initial flow images demonstrate tracer activity in bilateral extracranial carotid arteries and minimal intracranial tracer activity. Delayed static images of the brain demonstrate activity in the superior sagittal (dashed black arrows in (**C**,**D**)) and bilateral lateral sinuses (solid black arrows in (**D**)), representing venous circulation. These findings do not satisfy the criteria for a complete lack of cerebral perfusion.

Initial flow images are acquired at 1–2 s per frame for at least 60 s. Image acquisition starts before or while injecting the tracer to confirm that the imaging that started before the bolus reaches the carotid arteries and image acquisition ends after the venous phase. Initial flow images are essential for interpreting non-brain binding agents (^99m^Tc-DTPA), which usually demonstrate tracer flow in intracranial circulation. Sometimes, while using brain binding or parenchymal perfusion agents (^99m^Tc-HMPAO and ^99m^Tc-ECD), the suboptimal visualization of the brain on delayed images caused by the improper preparation or instability of the radiopharmaceutical may occur. In those situations, flow images will help to confirm a lack of brain blood flow when the brain is not visualized on delayed images. For the brain binding agents, 20 min delayed static planar images are acquired in the anterior, posterior, and lateral views. Additionally, SPECT images can be obtained for brain binding agents, but it is practically not feasible for unstable patients [35]. Flow images show the tracer flow in bilateral carotids and bilateral middle and anterior cerebral arteries in patients with intact blood flow (Figure 1). In BD/DNC, there is the absence of intracranial circulation with tracer activity in the nose region in anterior projection due to the rerouted blood flow to the region of the brain stem and cervical spinal cord called a “hot nose” sign [36]. Caution should be taken not to mistake scalp external carotid circulation for intracranial circulation [37]. 

**Figure 2:**
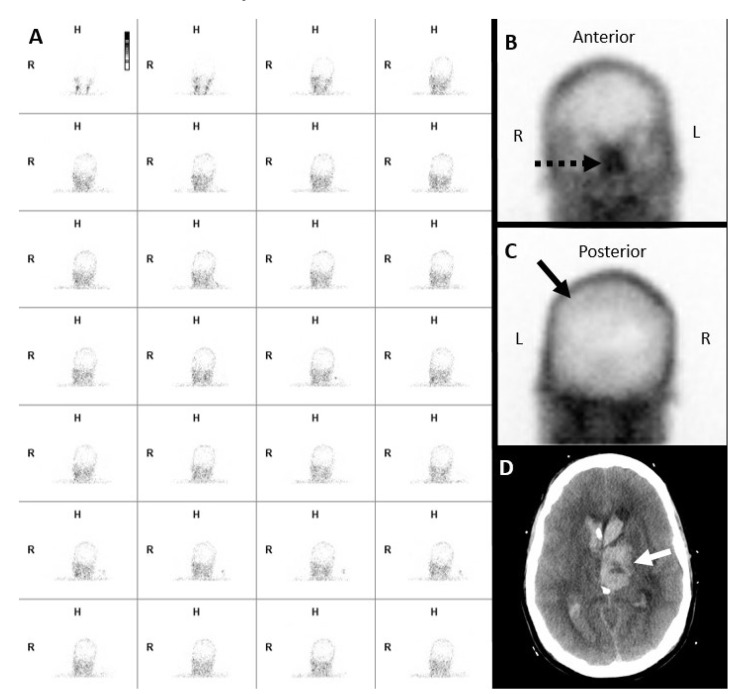
Forty-four-year-old female. A Tc-99m DTPA nuclear scintigraphy study after the injection of 25.5 mCi of Tc-99m DTPA. (**A**) Initial blood flow phase image series at 2 s intervals. (**B**,**C**) Delayed static images at 20 min after tracer injection in (**B**) anterior and (**C**) posterior projections. Initial flow images demonstrate tracer activity in bilateral extracranial carotid arteries without intracranial tracer activity. Delayed static images of the brain demonstrate a lack of tracer activity in the supra and infratentorial brain parenchyma. These findings satisfy the criteria for a complete lack of cerebral perfusion. Noted are the “hot-nose sign” (dashed black arrows in (**B**)) and “empty light-bulb sign” (solid black arrows in (**C**)). (**D**) Unenhanced axial CT brain in brain window demonstrates acute parenchymal hemorrhage in left basal ganglia (solid white arrow) with the intraventricular extension of hemorrhage with the associated mass effects and midline shift.

**Figure 3:**
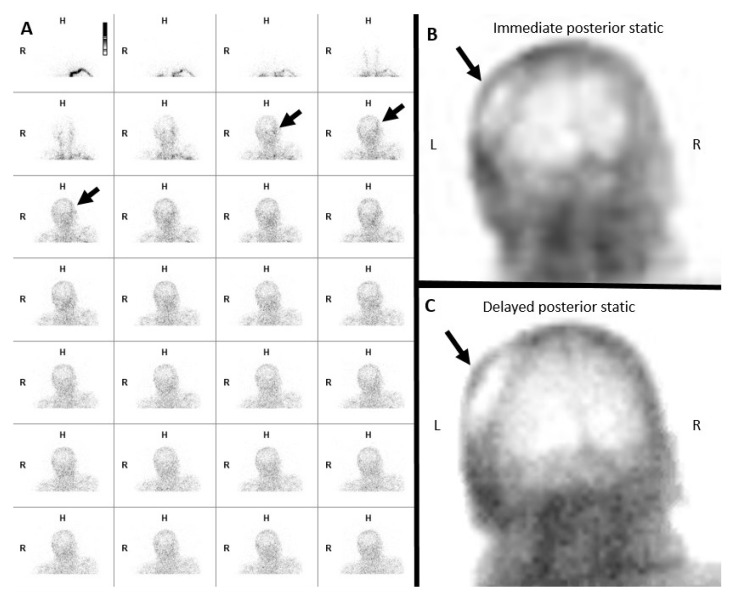
False negative nuclear brain scintigraphy study. A sixty-two-year-old male, post-surgical evacuation of left cerebral convexity subdural hematoma. A Tc-99m DTPA nuclear scintigraphy study after the injection of 27.5 mCi of Tc-99m DTPA. (**A**) Initial blood flow phase image series at 2 s intervals. (**B**) Immediate static and (**C**) 30 min delayed static images in the posterior projections. Initial flow and static images demonstrate tracer activity in the left scalp (black arrows in (**A**–**C**)), limiting evaluation of the absence of intracranial tracer activity.

In the patient with intact blood flow to the brain, superior sagittal sinus flow is seen in the venous phase of the study. Absent or minimal tracer activity in the superior sagittal sinus with the absent flow in carotids and intracranial arteries is sometimes seen in BD/DNC patients. However, the mere presence of activity in the superior sagittal sinus in the absence of demonstrable cerebral arterial flow activity was not clinically significant and did not contradict the diagnosis of BD/DNC, according to a retrospective study by V W Lee et al. [38]. Intracranial pressure transducers and cerebrospinal fluid shunts cause hyperemia in the scalp and yield false negative BD/DNC studies. Pressure on the posterior scalp during scans due to a hard surface and the post-traumatic disruption of the skull and scalp cause relative photogenic regions on the flow phase, yielding a false decrease in the cerebral flow. In BD/DNC during the delayed phase with brain-specific agents, there is no tracer uptake in the brain parenchyma, producing a “hollow skull” or “empty bulb” sign (Figure 2). In patients with a head injury, false tracer activity in the superior sagittal sinus region is due to the hyperemic blood flow to the injured scalp, which yields a false negative BD/DNC study [28] (Figure 3). The complete study includes an evaluation of cerebral hemispheres and the cerebellum; therefore, planar scintigraphy is performed in anteroposterior or posteroanterior views with at least one lateral projection [32].

### 7.2. Digital Subtraction Angiography (DSA) 

Another gold standard for ancillary imaging tests is DSA, which determines the cerebral blood flow. The catheter tip is advanced to the aortic arch, and contrast is injected into each of the four arteries supplying the brain [39]. Furthermore, 10 mL of 300 mg/mL of the iodinated nonionic contrast is injected at an 8 mL/second rate for the common carotids and a 5 mL/second rate for the selective vertebral arteries injection [29]. The selective injection of the common carotid and vertebral arteries is preferred over the aortic arch injection [39]. 

**Figure 4:**
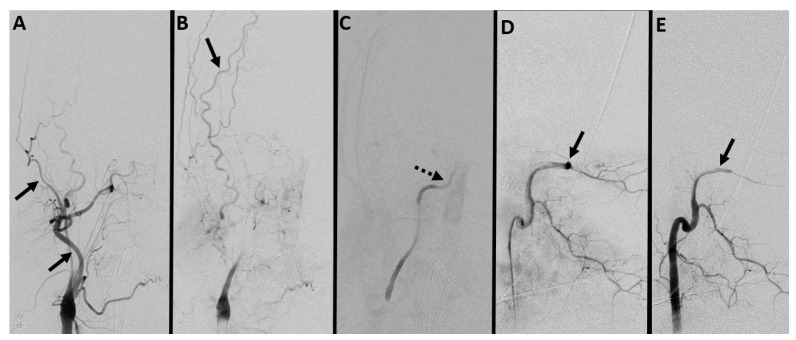
Digital subtraction angiography (**A**–**C**) right carotid and (**D**,**E**) vertebral artery. Lack of visualization of ICA beyond the petrous segment (dashed black arrow in (**C**)) and vertebral artery beyond the intradural segment (solid black arrows in (**D**,**E**)) with the normal contrast opacification of the right external carotid artery and its branches (solid black arrows in (**A**,**B**)). (Case courtesy—Dr. Anne Osborn).

**Figure 5:**
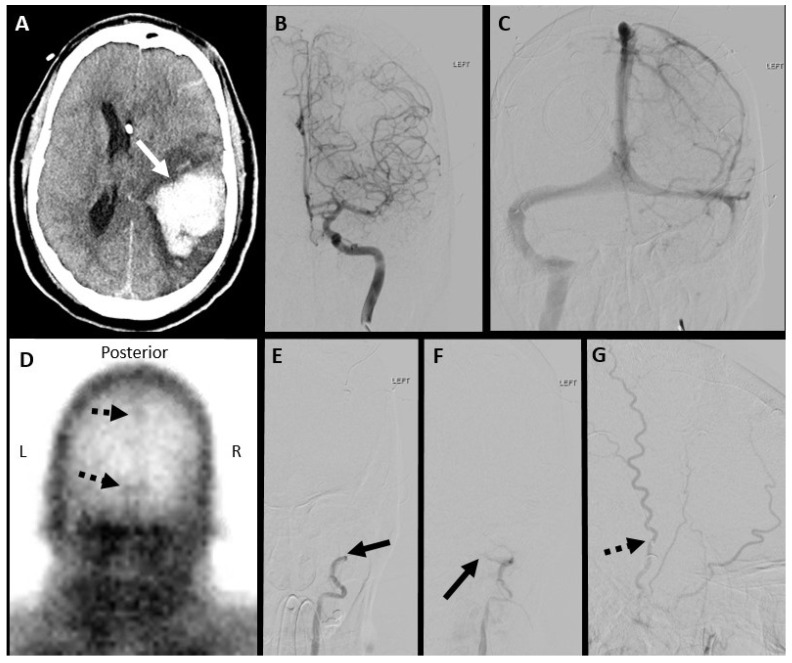
Fifty-four-year-old female with BD/DNC in the clinical deterioration of intracranial hemorrhage. (**A**) Unenhanced axial CT brain at the initial presentation demonstrates parenchymal hemorrhage in the left fronto-parietal lobe with mass effect (solid white arrow) with associated subarachnoid hemorrhage. (**B**) Arterial and (**C**) venous phase of carotid angiogram at the initial presentation does not demonstrate active contrast extravasation with patent intracranial arterial and venous circulation. Patient clinically deteriorated over a period of 7 days with clinically absent brain stem reflexes. (**D**) Faint intracranial midline tracer activity on Tc-99m DTPA nuclear scintigraphy favoring the inconclusive diagnosis of the BD/DNC (dashed black arrows in (**D**)). (**E**–**G**) Follow-up catheter angiography was performed, and it confirms the diagnosis of BD/DNC where nuclear scintigraphy was inconclusive. There is the non-visualization of internal carotid artery beyond the petrous segment (solid black arrows in (**E**,**F**)) with the contrast opacification of branches of external carotid arteries (dashed black arrow in (**G**)).

At least 20 s of biplane, anteroposterior, and lateral acquisition is performed to limit the radiation exposure and quantity of contrast. In a patient with BD/DNC, an absence of intracerebral contrast opacification in the internal carotid artery beyond the petrous segments and intradural bilateral vertebral arteries with normal external carotid arteries is observed. An absence of contrast opacification in internal cerebral veins and the vein of Galen is also noted. There may be the delayed contrast opacification of the superior sagittal sinus [29] (Figure 4 and Figure 5). At least two injections, 20 min apart, must show an absence of the filling of four arteries as their course becomes intracranial [39,40].

The drawback of the procedure is that it is expensive, invasive, and requires an expert operator to perform it. Iodinated contrast carries the potential risk of renal injury, especially if organ transplantation is a consideration. DSA yields false positives in hypotensive patients. Furthermore, “stasis filling” can occur due to the diffusion of contrast in the static column of blood, which can result in false negatives. Decreased intracranial pressure in the status post craniotomy, post ventricular drain, and open skull fracture also yield false negative DSA for BD/DNC [41].

### 7.3. Transcranial Doppler (TCD)

**Figure 6:**
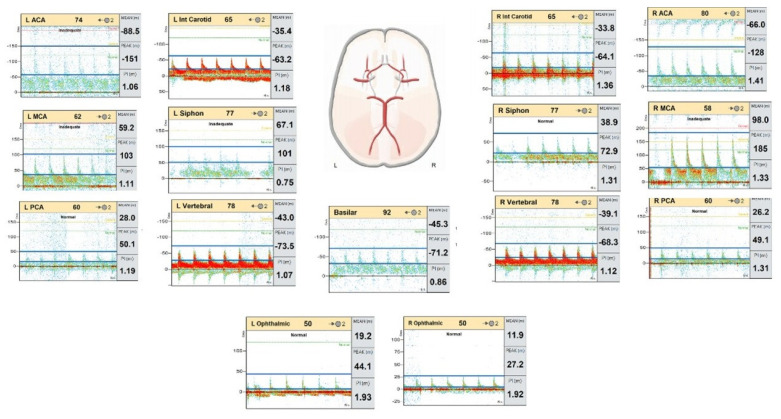
Normal transcranial Doppler study in 54-year-old female with intracranial hemorrhage. Normal antegrade flow direction in the bilateral internal carotid, carotid siphon, ACA, MCA, PCA, vertebral, basilar, and Ophthalmic arteries with maintained velocity. No evidence of vasospasm. No evidence of diastolic reversal or absent diastolic flow. (Flow sheet courtesy: VIASONIX).

TCD ultrasound provides rapid, noninvasive, operator-dependent, real-time measures of cerebrovascular function. TCD is cheaper, safer, and readily available. TCD can be used to measure the flow velocity in the brain’s basal arteries to assess relative flow changes. According to Monteiro et al., TCD is 89% sensitive and 99% specific for diagnosing BD/DNC [42]. A 2 MHz probe is used for TCD. To evaluate the middle cerebral arterial waveform, the transducer is placed in the bitemporal window above the level of the zygomatic arch. And for the evaluation of the vertebrobasilar arterial waveform, the transducer is placed posteriorly in the suboccipital window. Typically, antegrade flow is observed in the bilateral intracranial cerebral arteries (Figure 6). Reverberating, absent, or oscillatory flow are diagnostic for BD/DNC. For confirmation, there is a need to perform two examinations 30 min apart [29]. False negative TCD results are seen in post-craniotomy patients, and TCD does not directly evaluate the brain stem function [43]. According to Riggs et al., the central retinal artery Doppler can be considered as an ancillary test in the pediatric patient through an eye window [44]. 

### 7.4. Computed Tomography (CT) Brain

**Figure 7:**
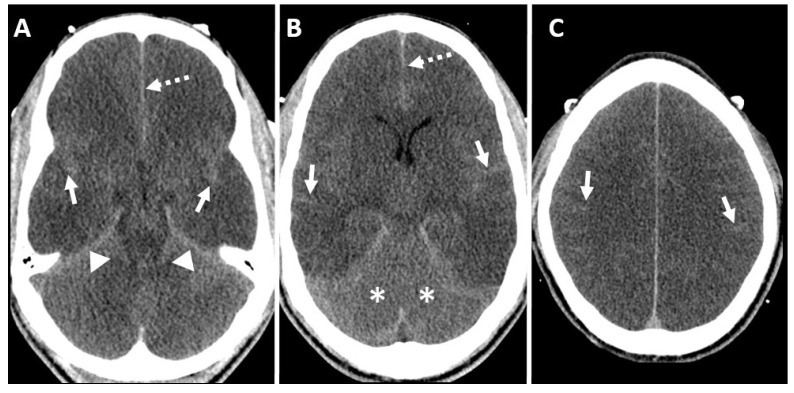
Unenhanced axial CT brain (**A**) at the level of the temporal lobe, (**B**) at the level of basal ganglia, and (**C**) at the level of centrum semiovale demonstrate diffuse cortical edema in supratentorial brain parenchyma with blurring of the gray-white matter interface with increased attenuations of the sulci (solid white arrows in (**A**–**C**)), interhemispheric falx cerebri (dashed white arrow in (**A**,**B**)) and bilateral tentorial cerebri (solid white arrow heads in (**A**)), resulting in a false appearance of subarachnoid hemorrhage also called “Psuedosubarchnoid hemorrhage”. There is a slight hyperdense appearance of the cerebellum (white stars in (**B**)) called “white cerebellum” or “cerebellar reversal sign”.

**Figure 8:**
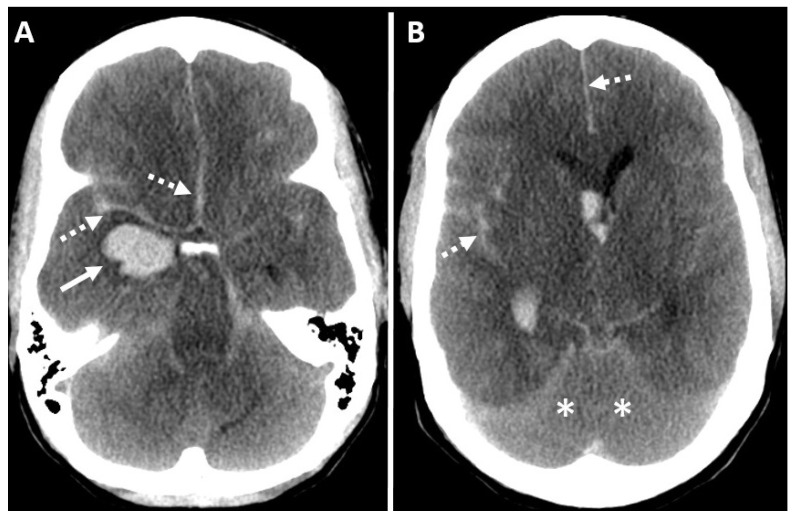
Unenhanced axial CT brain (**A**) at the level of the temporal lobe and (**B**) at the level of basal ganglia in a patient with cocaine abuse demonstrate right temporal lobar hemorrhage (solid white arrow in (**A**)), diffuse subarachnoid hemorrhage (dashed white arrows in (**A**,**B**)), and intraventricular hemorrhage. Supratentorial parenchymal hypodensity with hyperdense appearance of the cerebellum (white stars in (**B**)) called “white cerebellum” or “cerebellar reversal sign”.

**Figure 9:**
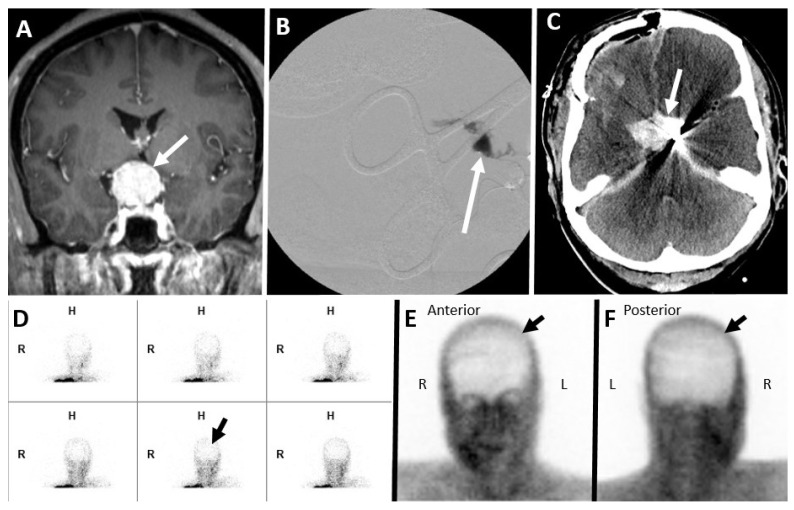
Forty-nine-year-old female with suprasellar mass lesion. (**A**) Coronal T1 post contrast image shows enhancing suprasellar mass lesion (white arrow in (**A**)). Patient underwent surgical resection and developed intra-operative massive bleeding. (**B**) Intra-operative fluoroscopy image shows contrast extravasation from avulsed cavernous left internal carotid artery, and the patient underwent embolization and repair for the same issue. (**C**) Axial unenhanced CT image of the brain shows hematoma in the basal cistern (white arrow) with streak artifact from the coil embolization of left internal carotid artery with post-surgical change. Tc-99m DTPA nuclear scintigraphy study, (**D**) initial blood flow phase images, (**E**) static anterior, and (**F**) posterior images demonstrate the absence of intracranial tracer activity (black arrows in (**D**–**F**)), confirming BD/DNC. There is tracer activity in the right scalp from recent surgery.

CT is fast and readily available. A non-contrast CT head has 32.7% sensitivity and 73.3% specificity for diagnosing BD/DNC [45]. The diffuse loss of gray-white matter differentiation is one of the most important features of BD/DNC; however, this finding may appear late, and the inter-rater reliability is poor [45]. The diminution of ambient cistern visualization, >10 mm of midline shift, and grey matter/white matter ratio <1.18 predict the progression to BD/DNC. Supratentorial parenchymal hypodensity with the hyperdense appearance of the cerebellum called “white cerebellum” or “cerebellar reversal sign” may also be observed (Figure 7). Diffuse cortical edema in the supratentorial brain parenchyma, along with the blurring of the gray–white matter interface and increased attenuation of the sulci, can create a false appearance of subarachnoid hemorrhage, known as ‘pseudosubarachnoid hemorrhage’ (Figure 8). CT is not able to provide any functional or blood flow information. Contrast-enhanced CT of the head can be acquired; however, it is a non-reliable method because of the delayed imaging. This delay makes the contrast-enhanced CT highly susceptible to the “stasis filling” of the brain blood vessels. Non-contrast CT helps to detect the parenchymal hemorrhage. Hemorrhagic complications associated with transsphenoidal surgery are rare, but when they occur, they may lead to death or permanent disability [46,47] (Figure 9).

### 7.5. Computed Tomography Angiography (CTA) Brain

CTA was first reported in 1998 as an ancillary test in diagnosing BD/DNC [48]. CTA is readily available, fast, noninvasive, and inexpensive. In addition, CTA has a high spatiotemporal resolution and is relatively operator-independent. Several European countries have adopted CTA as an ancillary test, but not the United States [49,50,51]. A new national guideline from the UK, published in the journal *Anaesthesia* in January, offers recommendations on the use of cerebral computed tomography (CT) angiography (CTA) as an ancillary investigation in order to aid in the diagnosis of death by neurologic criteria (DNC) in specific situations [52,53]. The technique of CTA includes the rapid intravenous injection of 60 to 120 mL of iodinated contrast at a rate of 3 to 5 mL/second. The contrast can be followed by the infusion of 30 to 40 mL of normal saline at the same rate to push the contrast medium forward and to optimize the contrast enhancement. 

**Figure 10:**
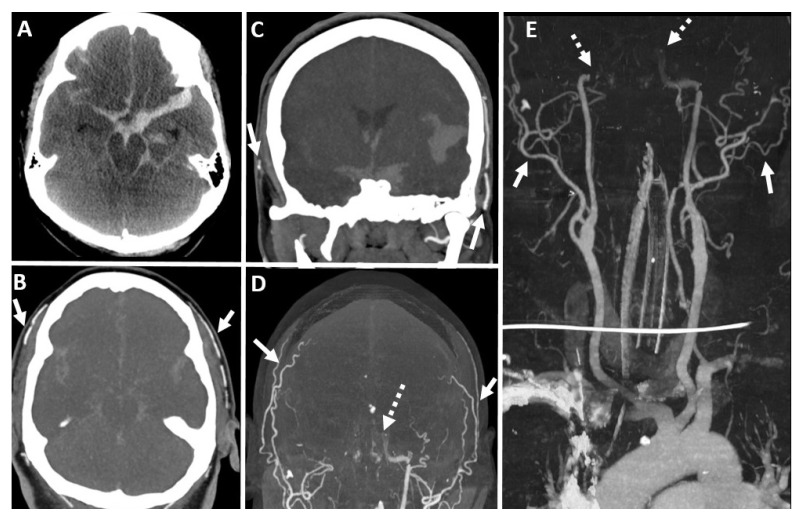
(**A**) Unenhanced axial CT brain image, (**B**) axial MIP (maximum intensity projection), (**C**) coronal MIP, (**D**) 3D coronal reconstruction of head, and (**E**) 3D coronal reconstruction of CT neck angiography after an injection of 80 mL contrast iopamidol injection 76% (Isovue 370, Bracco) in a patient with BD/DNC. There is diffuse subarachnoid hemorrhage with cerebral edema (**A**). CT head and neck angiography demonstrate the contrast opacification of bilateral external carotid arteries and its branches (solid white arrows in (**B**–**E**)). The contrast opacification of the bilateral internal carotid artery up to the petrous segments (dashed white arrows in (**D**,**E**)). The absence of the contrast opacification of intracranial carotid and vertebral arteries. There is an artifact from endotracheal tube in the reconstructed image of the neck.

At least three acquisitions should be performed for the diagnosis of BD/DNC. The first acquisition is a non-contrast scan as a baseline reference for assessing vascular opacification. The second acquisition is the early post-contrast phase, started approximately 20 s after the beginning of injection for the evaluation of intracranial and extracranial vascular opacification. The contrast opacification of the external carotid arteries indicates that the contrast is correctly delivered and rules out no associated hemodynamic abnormality (Figure 10). Advanced techniques like bolus tracking and test bolus can be used to tailor the contrast injection to the patient. The superficial temporal artery is used to assess sufficient contrast delivery. In cases where the surgical ligation of the superficial temporal artery is performed, such as following bilateral craniectomy, a good alternative is a facial artery, assessed for sufficient contrast delivery. The third acquisition is the late post-contrast phase, started 60 s after the injection for the evaluation of delayed intracranial vascular opacification, and helps to minimize false-positives from the early phase termination of the study in the absence of contrast opacification in the early phase.

The earliest sign of cerebral circulatory arrest in CTA is the lack of the opacification of the internal cerebral vein (ICV) and great cerebral vein (GCV) with a sensitivity between 98 and 100% for the diagnosis of BD/DNC [54]. The absence of the contrast opacification of cortical branches of the middle cerebral arteries (MCA-M4), basilar artery, and cortical branches of the posterior cerebral arteries (PCA-P2) have 86–100%, 85–94%, and 79% sensitivity, respectively, for BD/DNC diagnosis. CTA has 69.7–100% sensitivity for diagnosing BD/DNC [28]. Diagnostic criteria for BD/DNC using CTA include a lack of intracranial arterial contrast opacification. It can be assessed by four-, seven-, and ten-point scales. On a four-point scale, MCA-M4 and ICV are evaluated for contrast opacification [48]. The seven-point scale includes the evaluation of MCA-M4, ACA (anterior cerebral artery), ICV, and GCV [55]. In the ten-point scale, all seven segments of the seven-point scale, plus PCA-P2 and the basilar artery, are included [40]. 

### 7.6. Computed Tomography Perfusion (CTP) Brain

CT perfusion provides anatomical as well as functional information about the brain. CTP is primarily used for the evaluation of cerebral ischemia. This technique can also help calculate the CBF and CBV. A normal CBF in the brain is 50–60 mL/100 mg/min, and the CTP can measure as low as 1.2 mL/100 mg/min [56,57]. A total of 40 mL of nonionic iodinated contrast medium is injected at the rate of 5 mL/seconds, followed by 40 mL of saline flush at the rate of 5 mL/seconds. Regular perfusion analysis is performed if intracranial arteries are seen on the source images [56]. There is no intracranial CBF or CBV in the supratentorial and infratentorial compartments in BD/DNC. CTP can be performed along with the CTA. According to Escudero et al., CTP is 89% sensitive to the diagnosis of BD/DNC [56]. CTP at the brain stem is 100% sensitive, compared to the 72.7% sensitivity from the CTA scores [56]. The drawback of CTP is the potential contrast exposure, considering future organ donation and the issue of radiation exposure in a terminally ill patient. 

### 7.7. Magnetic Resonance Imaging (MRI) Brain 

In the early 1990s, the first reports demonstrated the potential role of MRI and MR spectroscopy in diagnosing BD/DNC. Typical MRI findings in BD/DNC patients are variable edema, diffuse cortical high signal intensity, diffuse cerebral white matter injury, and tonsillar herniation [58,59]. 

MR echo-planar images show the absence of a normal vascular flow void. In a study by Sohn et al., the loss of the intra-arterial flow signal voids on T2-WI, and tonsillar herniation is highly sensitive and specific for diagnosing BD/DNC [60]. T2*WI GRE and SWI demonstrate bilateral transcerebral vein signs (branching structure extending through cerebral hemispheres parallel or perpendicular to the outer wall of a lateral ventricle) and bilateral cortical vein signs (visualization of the cortical veins of the cerebral hemisphere) in a patient with BD/DNC. 

**Figure 11:**
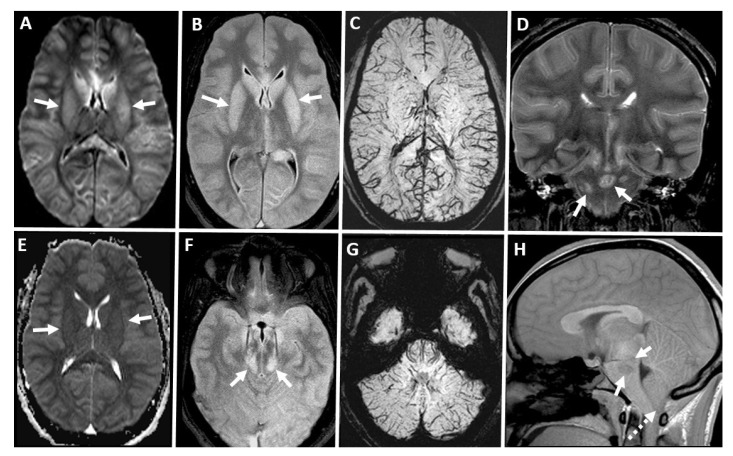
(**A**) Axial diffusion-weighted (repetition time msec/echo time msec, 8500/100; flip angle, 90°; b value = 1000 sec/mm^2^) and (**E**) corresponding axial apparent diffusion coefficient maps (8500/100; flip angle, 90°; b value = 1000 sec/mm^2^). (**B**,**F**) Axial FLAIR (fluid-attenuated inversion recovery; inversion time 2500, repetition time msec/echo time msec, 9000/107, 5 mm section thickness), (**C**,**G**) axial susceptibility weighted (repetition time msec/echo time msec, 28/20, 1.5 mm section thickness), (**D**) coronal T2 weighted (repetition time msec/echo time msec, 3711.1/93, 4 mm section thickness), and (**H**) sagittal T1-weighted unenhanced (repetition time msec/echo time msec, 250/2.48, 5 mm section thickness). MRI images of the brain in a patient with BD/DNC. There is restricted diffusion with FLAIR high signals in bilateral caudate and putamen deep nuclei (solid white arrows in (**A**,**B**,**E**)) with increased cortical SWI venous signals in supra and infratentorial brain parenchyma (**C**,**D**). Associated descending tentorial and tonsillar herniation (dashed white arrow in (**H**)) with durate hemorrhages in bilateral mid brain and pons (solid white arrows in (**D**,**F**,**H**)).

Diffusion-weighted imaging (DWI) and apparent diffusion coefficient (ADC) mapping can identify areas of cytotoxic damage and ischemic damage [54]. DWI may provide additional information on the brain tissue injury, but is not a practicable confirmatory test for the reliable diagnosis of BD/DNC due to the effects of the pseudo normalization of ADC values 5–28 days after ischemic brain damage [61] (Figure 11). The complete absence of adenosine triphosphate and the domination of the intense inorganic phosphate signal can be seen using P-31 MR-spectroscopy [62]. fMRI (functional MRI) of a brain with blood oxygen level-dependent signals shows no significant functional connectivity in a brain-dead patient, in contrast to a vegetative-state patient with preserved cortico-cortical connectivity [63]. The length of the scan and the availability of MRI are also some limiting factors, apart from the known MRI contraindications.

### 7.8. Magnetic Resonance Angiography (MRA) Brain

**Figure 12:**
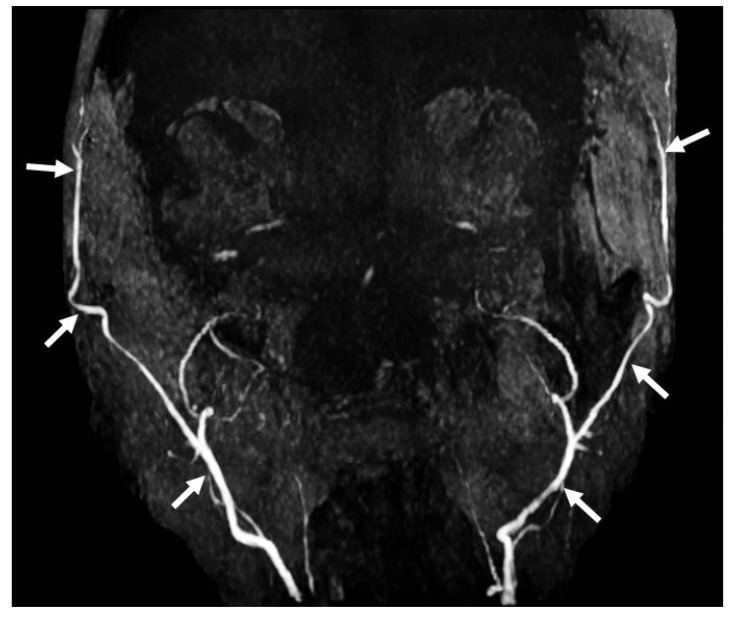
Seventy-six-year-old female with BD/DNC, 3D time-of-flight (TOF) MRI brain angiogram image shows an absence of flow in bilateral intracranial internal carotid arteries with normal flow signals in the bilateral external carotid artery and its branches (white arrows).

MRA is a reliable test for the cerebral blood flow [64]. MRA using the time-of-flight technique shows the absence of flow-related signals in the intracranial vessels [58] (Figure 12). Gadolinium-enhanced brain MRA demonstrates cerebral circulatory arrest when major intracranial vessels fail to fill with contrast, while extracranial vessels show a normal blood flow and contrast opacification [61]. Furthermore, 3T-MRA is 100% sensitive to the diagnosis of BD/DNC [60]. However, MRA is still not very reliable in assessing BD/DNC. Again, a longer scan time, difficulty in obtaining MRI on ventilated patients, availability, and magnetic contraindications are disadvantages of this modality.

### 7.9. Magnetic Resonance Perfusion (MR Perfusion) Brain

Apart from the blood flow, MR perfusion can detect the perfusion parameters of affected brain tissues, such as the cerebral blood flow, cerebral blood volume, and the absence of supratentorial and infratentorial brain perfusion. However, it has the same disadvantages as other MRI techniques and may be unreliable.

### 7.10. Positron Emission Tomography (PET) Brain

Absent metabolic activity with a “Hollow skull” appearance can be seen in a patient with BD/DNC [65], as reported in a few case reports. Since the brain uptake of the FDG (flurodeoxyglucose) PET tracer is affected by the blood glucose levels, decreased diffuse brain uptake may be seen in hypoglycemia; hence, PET imaging is unreliable in assessing BD/DNC. Therefore, PET is not a recommended imaging technique for the diagnosis of BD/DNC by any international society. 

## 8. Conclusions

BD/DNC is an irreversible cessation of brain functions, including the brainstem reflexes and circulatory and respiratory functions. The declaration of BD/DNC is associated with ethical and legal controversies. The early diagnosis of BD/DNC is important to prevent unnecessary medical interventions and expedite organ transplantation whenever possible. The AAN has already set the clinical criteria for diagnosing BD/DNC, including coma or unresponsiveness, apnea, and the absence of brainstem reflexes. Ancillary tests are required to support the diagnosis of BD/DNC when an apnea test or neurological examination cannot be performed. This review article will help clinicians and radiologists understand the role and limitations of various ancillary imaging tests in diagnosing BD/DNC.

## Figures and Tables

**Table 1:** Methods of ancillary testing for the determination of BD/DNC. This table is according to the 2023 guideline updates. Please note that electroencephalography is no longer considered an appropriate ancillary exam [5].

Ancillary Testing for the Determination of Brain Death/Death by Neurologic Criteria (BD/DNC)
**Catheter cerebral angiography** Inject the contrast medium into the aortic arch under high pressure to ensure it reaches both the anterior and posterior circulations. The external carotid circulation should be patent, and no intracerebral filling should be detected at the entry level of the carotid or vertebral artery into the skull. Additionally, the filling of the superior longitudinal sinus may be delayed.
**Transcranial Doppler ultrasonography** Transcranial Doppler (TCD) is effective only if a reliable signal is obtained, indicated by either reverberating flow or small systolic peaks in early systole. Bilateral insonation, including both anterior and posterior, is necessary. The probe should be positioned at the temporal bone above the zygomatic arch and at the vertebrobasilar arteries through the suboccipital transcranial window. Insonation through the orbital window can also be considered to obtain a reliable signal. However, a complete absence of flow may be unreliable due to inadequate transtemporal windows for insonation. Additionally, TCD may be less reliable in patients who have had a prior craniotomy.
**Cerebral scintigraphy (Technetium Tc-99m hexametazime—HMPAO)** The isotope should be administered within 30 min of the reconstitution. Both an anterior and lateral planar image of the head should be obtained at various time points as follows: immediately, between 30 and 60 min later, and at 2 h. Additional optional images of the liver demonstrating uptake can confirm a correct IV injection. There should be no radionuclide localization in the territories of the middle cerebral artery, anterior cerebral artery, or basilar artery in the cerebral hemispheres, which is known as the hollow skull phenomenon. Tracer should not be present in the superior sagittal sinus, although minimal tracer may come from the scalp.

**Table 2:** Comprehensive protocol for assessing the brain death/death by neurologic criteria (BD/DNC).

**Prerequisites (All to be Verified):** ❑ Ensure the patient is in a state of coma, with permanence and known cause. ❑ Align neuroimaging with the injury mechanism. ❑ Verify the absence of CNS depressant drug effects (if necessary, conduct a toxicology screen; if barbiturates have been administered, ensure serum levels are <10 μg/mL). ❑ Confirm the absence of residual paralysis (perform electrical stimulation if paralytics have been used). ❑ Ensure there is no evidence of severe acid-base, electrolyte, or endocrine abnormalities. ❑ Maintain normothermia or mild hypothermia (core temperature >36 degrees Celsius for at least 24 h if hypothermia is present). Lastly, ensure the systolic blood pressure is ≥100 mm Hg. **Examination (Minimum of 1 in Adults and 2 in Pediatrics):** ❑ SQUARE Unresponsiveness to visual, auditory, and tactile stimuli. ❑ Nonreactive pupils are induced by bright light. ❑ Absence of motor response to noxious stimuli in all four limbs (spinally-mediated reflexes are permissible). ❑ The absence of the corneal reflex. ❑ The absence of oculocephalic reflex is assessed only if C-spine integrity remains intact. ❑ If oculocephalic reflexes are absent or cannot be assessed, evaluation of the oculovestibular reflex is necessary. ❑ Absence of gag and cough reflexes. ❑ Lack of facial movement in response to noxious stimuli at the supraorbital nerve or temporomandibular joint. Patients below 6 months old exhibit no sucking/rooting reflexes.	**Apnea Testing (1 test in Adults, 2 in Pediatrics):** ❑ Ensure patient’s hemodynamic stability. ❑ Adjust the ventilator to maintain normocarbia (PaCO_2_ 35–45 mm Hg). ❑ Preoxygenate the patient with 100% FiO_2_ for more than 10 min, achieving a PaO_2_ > 200 mm Hg. ❑ Maintain patient’s oxygenation with a positive end-expiratory pressure (PEEP) of 5 cm of water. ❑ Administer oxygen via a suction catheter to the level of the carina at 6 L/min or attach a T-piece with continuous positive airway pressure (CPAP) at 10 cm H_2_O. ❑ Disconnect the ventilator. ❑ Confirm absence of spontaneous respirations. ❑ Draw arterial blood gas at 8–10 min; then reconnect the patient to the ventilator. ❑ Ensure arterial pH is within the range of 7.35–7.45. ❑ If PCO_2_ is ≥ 60 mm Hg or shows a 20 mm Hg rise from the normal baseline value, the apnea test is considered positive. ❑ Alternatively, if the apnea test is aborted, take appropriate action accordingly. **Ancillary testing (Only One to Be Performed, and Only if Clinical Exam/Apnea Testing is inconclusive or Cannot Be Completed):** ❑ Cerebral Angiogram ❑ HMPAO SPECT ❑ TCD Time of Death (DD/MM/YYYY): _____/______/______ Name of Physician and Signature: __________________________________

**Table 3:** Etiology and pathophysiology of brain death/death by neurologic criteria (BD/DNC). Abbreviations: ICP: Intracranial pressure; MAP: Mean arterial pressure.

Etiology of brain death:	Pathophysiology of brain death:
Intracranial causes Global: Diffuse cerebral edema Localized: Middle cerebral artery stroke etc. Hemorrhagic: Subarachnoid, intraventricular, subdural or epidural hemorrhage Traumatic brain injury	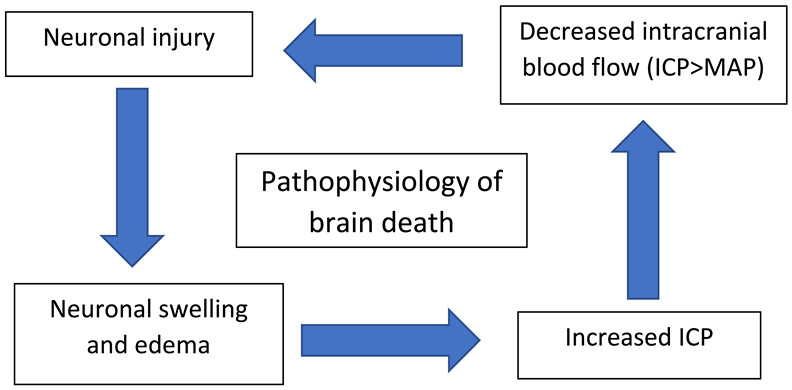
Extracranial causes Cardiopulmonary arrest without appropriate resuscitation -> decreased intracranial blood flow -> ischemic brain damage	

## Abbreviations

AAN	American Academy of Neurology
ACA	Anterior cerebral artery
ADC	Apparent diffusion coefficient
BBB	Blood–brain barrier
BD/DNC	Brain death/Death by neurologic criteria
CT	Computed tomography
CTA	Computed tomography angiogram
CTP	Computed tomography perfusion
CBF	Cerebral blood flow
CPP	Cerebral perfusion pressure
CVR	Cerebrovascular resistance
DSA	Digital subtraction angiography
DWI	Diffusion-weighted imaging
EEG	Electroencephalogram
fMRI	Functional brain MR
GSV	Great cerebral vein
GRE	Gradient-recalled echo
ICP	Intracranial pressure
ICV	Internal cerebral vein
MRI	Magnetic resonance imaging
MRA	Magnetic resonance angiogram
MAP	Mean arterial pressure
MR perfusion	Magnetic resonance perfusion
MAP	Mean arterial pressure
PET	Positron emission tomography
PaO_2_	Partial pressure of oxygen
PaCO_2_	Partial pressure of carbon dioxide
SPECT	Single-photon emission computerized tomography
SNM	Society of nuclear medicine
SWI	Susceptibility-weighted image
TCD	Transcranial Doppler
UDDA	Uniform Determination of Death Act
^99m^Tc-ECD	99m Technetium Ethyl cysteinate dimer
^99m^Tc-HMPAO	99m Technetium Hexamethylpropyelene amine oxime
^99m^Tc-DTPA	99m Technetium Diethylenetriaminepentaacetic acid
